# Absence of Spin‐Orbit Torque and Discovery of Anisotropic Planar Nernst Effect in CoFe Single Crystal

**DOI:** 10.1002/advs.202301409

**Published:** 2023-07-23

**Authors:** Qianbiao Liu, Xin Lin, Lijun Zhu

**Affiliations:** ^1^ State Key Laboratory for Superlattices and Microstructures Institute of Semiconductors Chinese Academy of Sciences Beijing 100083 China; ^2^ College of Materials Science and Opto‐Electronic Technology University of Chinese Academy of Sciences Beijing 100049 China

**Keywords:** Planar Nernst effect, spin polarization, spin current, spin orbit torque

## Abstract

Exploration of exotic spin polarizations in single crystals is of increasing interest. A current of longitudinal spins, the so‐called “Dresselhaus‐like” spin current, which is forbidden in materials lacking certain inversion asymmetries, is implied to be generated by a charge current at the interface of single‐crystal CoFe. This work reports unambiguous evidence that there is no indication of spin current of any spin polarizations from the interface or bulk of single‐crystalline CoFe and that the sin2*φ* second harmonic Hall voltage, which is previously assumed to *signify* Dresselhaus‐like spin current, is not related to any spin currents but rather a planar Nernst voltage induced by a longitudinal temperature gradient within the sample. Such sin2*φ* signal is independent of large applied magnetic fields and interfacial spin‐orbit coupling, inversely correlated to the heat capacity of the substrates and overlayers, quadratic in charge current, and appears also in polycrystalline ferromagnets. Strikingly, the planar Nernst effect (PNE) in the CoFe single crystal has a strong fourfold anisotropy and varies with the crystalline orientation. Such strong, anisotropic PNE has widespread impacts on the analyses of a variety of spintronic experiments and opens a new avenue for the development of PNE‐based thermoelectric battery and sensor applications.

## Graphical abstract

In CoFe single crystal an anisotropic planar Nernst effect is discovered, but there is no spin torque generated from the interface or bulk of the CoFe. The sin2*φ* dependent second harmonic Hall voltage signal in magnetic materials is not related to any generation of Dresselhaus‐like spin currents but rather a planar Nernst voltage.

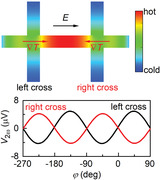

## 1 Introduction

Spin currents have the potential to efficiently manipulate magnetization via spin‐orbit torques (SOTs) in magnetic memory and computing technologies.^[^ ^1^, ^2^, ^3^, ^4^, ^5^ ^]^ The polarization vector ***σ*** of a spin current, in general, has three orthogonal components, that is, longitudinal (*σ_x_*), transverse (*σ_y_*), and perpendicular spins (*σ_z_*). A spin current of transverse spins, which can be efficiently generated by the spin Hall effect and other spin‐orbit effects,^[^ ^1^, ^2^, ^3^, ^4^, ^5^, ^6^, ^7^, ^8^, ^9^, ^10^ ^]^ can switch a transverse magnetization but not perpendicular and longitudinal magnetizations without the assistance of, for example, a magnetic field.^[^ ^11^ ^]^ While the exotic perpendicular (longitudinal) spins may drive magnetic‐field‐free switching of uniform perpendicular (longitudinal) magnetization directly or in combination with transverse spins, they are forbidden in conventional materials lacking inversion symmetry breaking.^[^ ^12^, ^13^ ^]^

Recently, exotic spins have been suggested in single‐crystal bulk^[^ ^14^, ^15^, ^16^ ^]^ and interfaces^[^ ^17^, ^18^, ^19^, ^20^ ^]^ with reduced symmetry in the lattice, Berry curvature, or magnetic configurations.^[^ ^21^, ^22^, ^23^, ^24^, ^25^, ^26^, ^27^, ^28^, ^29^ ^]^ While the generation efficiency is still very low (typically 2–3 orders of magnitude lower than transverse spins in Pt),^[^ ^20^ ^]^ exotic spins have stimulated considerable investigation^[^ ^14^, ^15^, ^16^, ^17^, ^18^, ^19^, ^20^ ^]^ and become a focused problem in the field of spintronics. Of particular surprise is a recent argument of longitudinal spins, Dresselhaus‐like spin current, from the interface of single‐crystalline CoFe samples based on a sin2*φ* contribution in the second harmonic Hall voltage (*V*_2ω_).^[^ ^30^ ^]^ This is very stimulating particularly when there have been many other reports of exotic spin currents and SOTs within single crystals.^[^ ^14^, ^15^, ^16^, ^17^, ^18^, ^19^, ^20^ ^]^ While the symmetry breaking that allows the longitudinal spins in that work^[^ ^30^ ^]^ was attributed to the low‐symmetry point group of the single crystal but not clearly specified, there have been other reports that the anisotropic magnetoresistance (AMR) and magnetic damping in single‐crystalline CoFe vary with the crystalline orientation,^[^ ^31^, ^32^ ^]^ implying a possibility to generating low‐symmetry and anisotropic effects in the same material.

In this work, we perform a systematic spin‐orbit torque study and unambiguously demonstrate that there is no indication of unbalanced spins of any polarizations from the interface or bulk of the CoFe single crystal. Contrary to the interpretation of the previous report,^[^ ^30^ ^]^ the sin2*φ* dependent *V*_2ω_ signal is *not* related to any generation of Dresselhaus‐like spin currents but rather a planar Nernst voltage (*V*_PNE_) induced by a longitudinal temperature gradient (∇*T_x_*) within the sample. Strikingly, we also identified that the planar Nernst effect (PNE) in CoFe single crystal is strongly anisotropic due to an intrinsic band crossing and varies in strength with the crystalline orientation of single crystals.

## 2 Sample Details and Measurement Techniques

For this work, single layers of 4 nm CoFe (CoFe = Co_50_Fe_50_) were grown on MgO (001) and GaAs (001) substrates, respectively, by the molecular beam epitaxy (MBE). Control samples include bilayers of CoFe 4 nm/Pt 4 nm grown on MgO (001) and GaAs (001) substrates by MBE and bilayers of MgO 2 nm/Py 9.4 nm, MgO 2 nm/Co 9.3 nm, and MgO 2 nm/FeCoB 9.6 nm sputter‐deposited on oxidized silicon substrates (FeCoB = Fe_60_Co_20_B_20_, Py = Ni_81_Fe_19_). Each sample is protected by a MgO 2 nm/Al 2 nm bilayer (MBE samples) or a MgO 1.6 nm/Ta 1.6 nm bilayer (sputtering samples) that were fully oxidized upon exposure to the atmosphere. All samples were grown and measured at room temperature.

**Figure 1:**
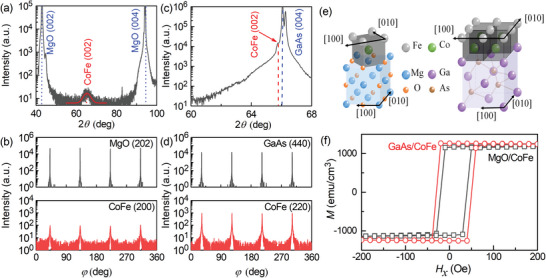
Structural characterizations. a) X‐ray diffraction *θ–*2*θ* pattern and b) *φ* scan patterns of the 4 nm thick CoFe films grown on MgO (001) substrate. c) X‐ray diffraction *θ–*2*θ* pattern and d) *φ* scan patterns of the 4 nm thick CoFe films grown on GaAs (001) substrate. e) Schematic of epitaxial relations for MgO (001)/CoFe and GaAs (001)/CoFe. (f) Magnetization hysteresis loops for the CoFe films grown on MgO (001) and GaAs (001) substrates.

X‐ray diffraction *θ*−2*θ* scan pattern in **Figure** **1**a and the fourfold *φ* scan patterns in Figure 1b indicate that the CoFe grown on MgO(001) is a single‐crystalline film with a body‐centered cubic (bcc) structure and epitaxial relation of MgO(001)[110]||CoFe(001)[100]. The CoFe lattice is rotated by 45^°^ in the film plane relative to the MgO lattice for the best lattice matching. This is because the lattice constant of the CoFe (≈0.286 nm in bulk but 0.285 nm in the film as indicated by the bcc (002) peak at 65.4^°^) is about $\sqrt{2}/2$ times that of MgO (≈ 0.421 nm). X‐ray diffraction *θ*−2*θ* pattern in Figure 1c and the fourfold *φ* scan patterns in Figure 1d indicate that the CoFe grown on GaAs (001) is a bcc single crystal with an epitaxial relation of GaAs(001)[110]||CoFe(001)[110]. The CoFe lattice has no in‐plane rotation relative to the GaAs lattice because the lattice constant of GaAs (0.5656 nm) is about two times that of the CoFe (≈0.284 nm in the film as indicated by the bcc (002) peak at 65.7^°^). Since accurate identification of the CoFe crystalline orientations is critical for the anisotropy analyses below, we further specify the epitaxial relation of the CoFe layers to the substrates using the schematics in Figure 1e.

**Figure 2:**
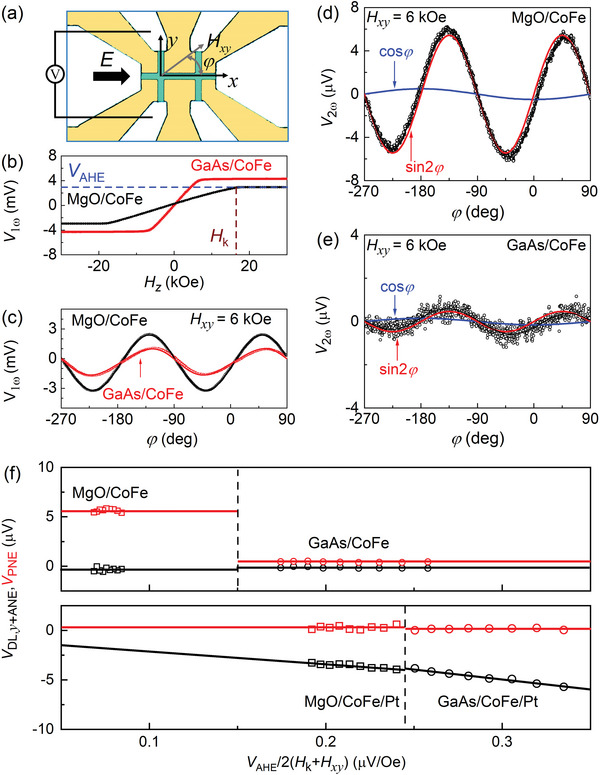
Harmonic Hall voltage results for the MgO/CoFe and GaAs/CoFe with the current flow along CoFe [110]. a) Optical microscopy image of the device structure and the measurement geometry. b) First harmonic Hall voltage (*V*_1_ *_ω_*) versus the perpendicular magnetic field (*H*_z_), c) *V*_1_ *_ω_* versus the in‐plane angle (*φ*) of the magnetic field (*H_xy_*) relative to the current direction (*H_xy_* = 6 kOe), d,e) *V*_2ω_ versus *φ* for the MgO/CoFe and GaAs/CoFe. f) Dependence of the planar Nernst voltage *V*_PNE_ and *V*_DL,_ *_y_*_+ANE_ on −*V*_AHE_/2(*H*_k_ + *H_xy_*) for MgO/CoFe, GaAs/CoFe, MgO/CoFe/Pt, and GaAs/CoFe/Pt. Solid curves in (c) represent the fit of the data to Equation (1); the solid black curves in (d) and (e) represent fits of the data to Equation (2), and the red and blue curves plot the sin2*φ* and cos*φ* components, respectively; solid lines in (f) represent linear fits of the data. For the data in (b–f), the applied electric field *E* is 6.67 × 10^4^ V cm^−1^.

Superconducting quantum interference device measurements indicate that all the CoFe samples in this study have in‐plane magnetic anisotropy and similar saturation magnetization (*M*_s_) regardless of the substrate and capping layer (*M*_s_ is 1380 emu cm^−3^ for the MgO/CoFe samples, 1220 emu cm^−3^ for the GaAs/CoFe samples, see Figure 1f for the hysteresis loops for the MgO/CoFe and the GaAs/CoFe). The samples were then patterned into two‐cross (5 × 60 µm^2^, **Figure** **2**a) or single‐cross (5 × 15 µm^2^) Hall bar devices with the current channel along different crystal orientations using photolithography and ion milling, followed by deposition of Ti 5 nm/Pt 150 nm as the electrodes for harmonic Hall voltage (HHV) measurements.

### 2.1 Absence of Spin‐Orbit Torques of Any Spin Polarizations

The strengths of the SOTs are qualified using HHV technique with the coordinates and geometry in Figure 2a. While applying a sinusoidal electric field (*E*), and thus current density *j* = *Eρ_xx_*, onto the bar orientated along the *x* direction, the in‐phase first and out‐of‐phase second HHVs, *V*_1ω_, and *V*_2ω_, are collected as a function of perpendicular magnetic field (*H*_z_) or the angle (*φ*) of the in‐plane magnetic field (*H_xy_*) relative to the current direction for different magnitudes of in‐plane magnetic field (*H_xy_* = 2–6 kOe). Here, *H_xy_* is chosen to be sufficient to make the magnetization colinear with it but not too large to introduce sizable ordinary Nernst voltage. When a *φ* ‐independent spin current with spin polarization (*σ_x_*, *σ_y_*, *σ_z_*) interacts with the macrospin,^[^ ^6^, ^7^, ^33^, ^34^, ^35^, ^36^, ^37^, ^38^ ^]^

(1) $$V_{1\omega }=V_{\mathrm{PHE}}\sin2\varphi$$

(2) $$\begin{array}{ccc} V_{2\omega } & = & V_{\mathrm{DL},x}\sin\varphi +V_{\mathrm{FL},x+\mathrm{Oe}}\sin\varphi \cos2\varphi +V_{\mathrm{DL},y+\mathrm{ANE}}\cos\varphi \\ & & +\;V_{\mathrm{FL},y+\mathrm{Oe}}\cos\varphi \cos2\varphi +V_{\mathrm{DL},z}\cos2\varphi +V_{\mathrm{PNE}}\sin2\varphi \\ & & +\;V_{\mathrm{FL},z+\mathrm{Oe}} \end{array}$$

with

(3) $$V_{\mathrm{DL},x}=V_{\mathrm{AHE}}/2\left(H_{k}+H_{xy}\right)$$

(4) $$V_{\mathrm{FL},x+\mathrm{Oe}}=-V_{\mathrm{PHE}}H_{\mathrm{FL},x}/2H_{xy}$$

(5) $$V_{\mathrm{DL},y+\mathrm{ANE}}=V_{\mathrm{AHE}}H_{\mathrm{DL},y}/2\left(H_{k}+H_{xy}\right)+V_{\mathrm{ANE}}$$

(6) $$V_{\mathrm{FL},y+\mathrm{Oe}}=-V_{\mathrm{PHE}}H_{\mathrm{FL},y+\mathrm{Oe}}/2H_{xy}$$

(7) $$V_{\mathrm{DL},z}=-V_{\mathrm{PHE}}H_{\mathrm{DL},z}/2H_{xy}$$

(8) $$V_{\mathrm{FL},z+\mathrm{Oe}}=V_{\mathrm{AHE}}H_{\mathrm{FL},z+\mathrm{Oe}}/2\left(H_{k}+H_{xy}\right)$$

Here, *V*_DL,_ *_x_* is the contribution of damping‐like SOT field of *σ_x_* (*H*_DL,_ *_x_*), *V*_DL,_ *_y_*_+ANE_ is the sum of the voltage contribution (*V*_DL,_ *_y_*) of dampinglike SOT field of *σ_y_* (*H*_DL,_ *_y_*) and the anomalous Nernst voltage (*V*_ANE_) from an perpendicular thermal gradient (∇*T_z_*); *V*_DL,_ *_z_* is the contribution of dampinglike SOT field of *σ_z_* (*H*_DL,_ *_z_*), *V*_FL,_ *_x_*_+Oe_ is the contribution of field‐like SOT field of *σ_x_* (*H*_FL,_ *_x_*) and longitudinal Oersted field;^[^ ^37^ ^]^ *V*_PNE_ is the planar Nernst voltage associated with the longitudinal temperature gradient (∇*T_x_*_)._ *V*_FL,_ *_y_*_+Oe_ is the contribution of the field‐like SOT field of *σ_y_* and the transverse Oersted field; *V*_FL,_ *_z_*_+Oe_ is the contribution of the field‐like SOT field of *σ_z_* (*H*_FL,_ *_z_*) and the perpendicular Oersted field.^[^ ^35^ ^]^ The anomalous Hall voltage (*V*_AHE_) and the effective magnetic anisotropic field (*H*_k_) of each sample are determined from the dependence of *V*_1ω_ on the swept *H_z_* under zero *H_xy_* (Figure 2b). Note that in this work *H*_k_ is defined as positive when the magnetic anisotropy is in the film plane. The values of *V*_PHE_ are extracted from fits of the *φ* dependence of *V*_1ω_ to Equation (1) (Figure 2c).

To check whether there were torques of any spin polarizations in the GaAs/CoFe and MgO/CoFe samples, we first measure the samples with the current flow along the CoFe [110] direction. As shown in Figure 2d,e, the best fits of the *V*_2ω_ data to Equation (2) yield a sizable sin2*φ* signal (*V*_PNE_ ≠ 0), a cos*φ* signal (*V*_DL,_ *_y_* + *V*_ANE_ ≠ 0), and a *φ* independent term (*V*_FL,_ *_z_*_+Oe_ ≠ 0). The lack of sin*φ*, sin*φ*cos2*φ*, cos2*φ*, cos*φ*cos2*φ* terms (i.e., *V*_DL,_ *_x_* = *V*_DL,_ *_z_* = *V*_FL,_ *_x_*_+Oe_ = *V*_FL,_ *_y_*_+Oe_ = 0) indicates negligible dampinglike SOT of *σ_z_* and *σ_x_* and negligible transverse and longitudinal field‐like torque (*H*_FL,_ *_x_*_+Oe_ = *H*_FL,_ *_y_*_+Oe_ = 0) in the CoFe samples. As revealed by the independence of *V*_DL,_ *_y_*_+ANE_ on *H_xy_* or −*V*_AHE_/2(*H*_k_ + *H_xy_*) (Figure 2f), there is also no dampinglike torque of *σ_y_* (*H*_DL,_ *_y_* = 0, see Equation (5)) in the GaAs/CoFe and MgO/CoFe samples. In contrast, in the same magnetic field range where −*V*_AHE_/2(*H*_k_ + *H_xy_*) is varied by 20%−25%, *V*_DL,_ *_y_*_+ANE_ does vary significantly in a linear manner with −*V*_AHE_/2(*H*_k_ + *H_xy_*) for the control samples GaAs/CoFe 4 nm/Pt and MgO/CoFe 4 nm/Pt that have transverse spins generated by the spin Hall metal Pt. We do not find any signal that is independent of *φ* but dependent on *H_xy_*, suggesting the absence of any field‐like torque of *σ_z_* or perpendicular Oersted field,^[^ ^35^ ^]^ that is, *H*_Oe,_ *_z_ = H*_FL,_ *_z_* = 0. Since the damping‐like torque of a spin current is typically greater than the field‐like torque,^[^ ^6^, ^7^, ^8^, ^9^, ^10^ ^]^ the negligible damping‐like torques of *σ_x_* and *σ_y_* most likely suggest negligible field‐like counterparts of *σ_x_* and *σ_y_* (*H*_FL,_ *_x_ = H*_FL,_ *_y_* = 0). Therefore, the longitudinal and transverse Oersted fields should also be negligible (as indicated by the zero *H*_FL,_ *_x_*_+Oe_ and *H*_FL,_ *_y_*_+Oe_). So far, we have excluded the generation of SOTs of any spin polarizations from the bulk or interface of the CoFe single crystal. Instead, we identify that the cos*φ* and sin2*φ* signals in Figure 2d,e are purely from thermoelectric effects, that is, the anomalous Nernst effect and the PNE.

We have also reaffirmed the above conclusions for the single‐crystalline GaAs/CoFe and MgO/CoFe samples when the current is rotated by ±30^°^, ±45^°^, ±60^°^, and ±90^°^ relative to the [110] direction of the CoFe crystal.

### 2.2 Origin of the sin2φ Second HHV Signal

In the above analysis, we have employed Equation (2) which assumes that the involved spin current is independent of *φ*, the orientation of the external field, and thus of the magnetization, relative to the current. However, in case the magnitude of the spin current was not constant but had a cos*φ* dependence due to some mechanism, longitudinal spins could also contribute a sin2*φ* second HHV signal. Note that a previous work^[^ ^30^ ^]^ had speculated that the sin2*φ* second HHV signals signified a “Dresselhaus‐like spin current” from the low‐symmetry point group of the interface of the single‐crystal CoFe. For a more unambiguous conclusion, we clarify below that the sin2*φ* second HHV signals can only be attributed to the PNE induced by a longitudinal thermal gradient.

**Figure 3:**
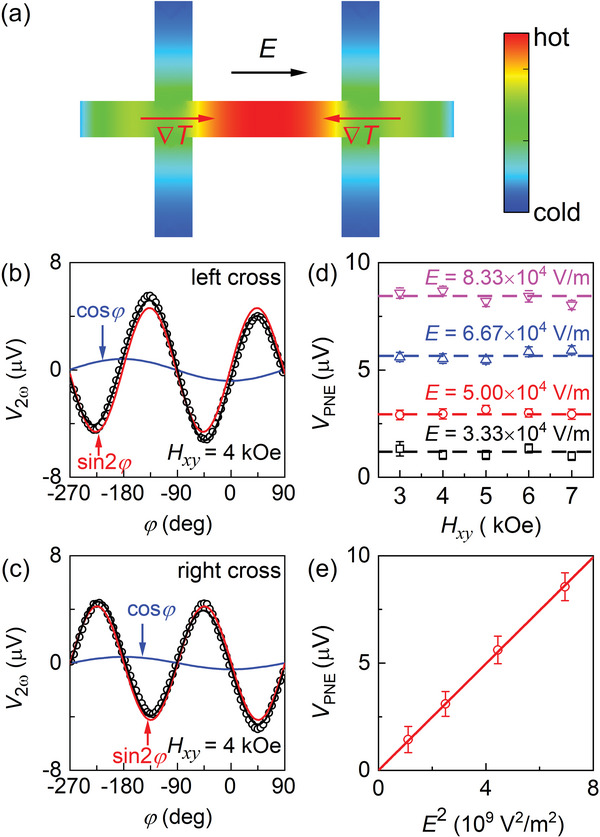
a) Simulated temperature non‐uniformity induced by current in a two‐cross Hall bar device. Dependence of *V*_2ω_ on the in‐plane angle (*φ*) of the magnetization relative to the electric field *E* for b) the left and c) the right Hall crosses of the “╫”‐shaped MgO/CoFe[110] Hall bar device, d) Planar Nernst voltage versus in‐plane magnetic field for the left cross of the MgO/CoFe[110] Hall bar as measured from under different electric field *E*. e) Linear dependence of the planar Nernst voltage on *E* ^2^. In (b) and (c), the solid black curves represent fits of the data to Equation (2), with the red and blue curves plotting the sin2*φ* and cos*φ* components, respectively. The dashed lines in (d) guide the eyes, and the solid line in (e) represents the best linear fit.

**Figure** **3**a shows the temperature non‐uniformity of the two‐cross Hall‐bar devices simulated using finite element analysis. For such “╫” shaped two‐cross Hall bar device, the temperature is higher at the center of the Hall bar device and lower in the region close to the big, thick electrodes which are good thermal sinks, resulting in in‐plane thermal gradients pointing from the Hall bar center to the electrodes. The in‐plane thermal gradients ∇*T_x_* that are collinear with the current are expected to induce a planar Nernst voltage (*V*_PNE_) that contributes to a sin2*φ* term to the second HHV signal.^[^ ^33^, ^35^, ^38^ ^]^ Exactly as expected, the two crosses of the Hall bar devices show strong sin2*φ* signals that are of similar magnitude but opposite signs (Figure 3b,c). (note that the cos*φ* signals are of the same sign for the two Hall crosses because they are the anomalous Nernst voltage induced by the *vertical* thermal gradient ∇*T_z_* that is of the same orientation for the two Hall crosses, see Equation (2) and discussions above). As shown in Figure 3d,e, the measured *V*_PNE_ scales with the applied electric field *E*, and thus the current density, in the CoFe layer in a parabolic manner whereas it is independent of the magnitude of the applied in‐plane magnetic field, reaffirming the nature of a thermoelectric effect.

**Figure 4:**
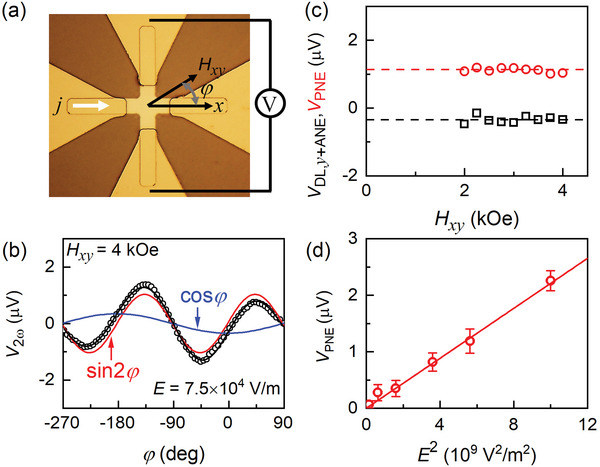
a) Optical microscopy image, b) Second harmonic signals (*V*_2ω_) versus the in‐plane angle (*φ*) of the magnetic field relative to the electric field, c) Independence of the planar Nernst voltage (*V*_PNE_) and *V*_DL,_ *_y_*_+ANE_ of *H_xy_*, and d) Linear dependence of *V*_PNE_ on *E* ^2^ for a single‐cross MgO/CoFe Hall device (5 × 15 µm^2^) that is nominally symmetric but exhibits unintentional symmetry imperfection. The solid black curves in (b) represent fits of the data to Equation (2) in the main manuscript, with the red and blue curves plotting the sin2*φ* and cos*φ* components; dashed lines in (c) guides the eyes; solid line in (d) represents linear fits of the data.

Such sin2*φ* signal can also show up in Hall crosses with unintentional symmetry imperfection (e.g., due to fabrication uncertainties during lithography and/or etching processes). In **Figure** **4**a–d we show the sin2*φ* signal measured from a nominally symmetric single‐cross MgO/CoFe Hall bar device. The sin2*φ* signal is independent of the applied in‐plane magnetic field and varies in linear proportion to *E* ^2^, in agreement with a thermoelectric effect. The sin2*φ* signal is only 20% of that of the two‐cross Hall‐bar devices but still significant. As we comment in Section S1 of the Supporting Information, the sin2*φ* signal in the previous study of CoFe samples^[^ ^30^ ^]^ also varies significantly with the location of the cross on the three‐cross Hall bar device, in agreement with the planar Nernst effect being the origin of the sin2*φ* signals. Note that the presence of effects due to an unintentional symmetry imperfection is common in microscale strips and crosses.^[^ ^35^, ^39^ ^]^ Two of the examples are non‐uniform current distribution effects in nominally symmetric Hall bars and spin‐torque ferromagnetic resonance microstrips^[^ ^35^ ^]^ and longitudinal temperature gradient‐induced planar Nernst signal in nominally symmetric NiFe Hall cross.^[^ ^39^ ^]^

Moreover, the value of *V*_PNE_ from the CoFe scales inversely with the thermal conductivity of the adjacent layers. Under the same electric field of *E* = 6.67 × 10^4^ V cm^−1^, *V*_PNE_ is ≈5.6 µV for MgO/CoFe 4 nm/MgO, 0.40 µV for GaAs/CoFe 4 nm/MgO, and 0.26 µV for MgO/CoFe 4 nm/Pt, and 0.19 µV for GaAs/CoFe 4 nm/Pt, which is in anti‐correlation to the relative magnitudes of their thermal conductivities (30 W mK^−1^ for MgO,^[^ ^40^ ^]^ 56 W mK^−1^ for GaAs,^[^ ^41^ ^]^ and 78 W mK^−1^ for Pt^[^ ^42^ ^]^).

In addition, the relative magnitude of the sin2*φ* second HHV signals, which is much weaker for the GaAs/CoFe than for the MgO/CoFe (**Table** **1**), is not consistent with the relative strength of the interfacial spin‐orbit coupling of the CoFe interfaces. Using the relation *H*_k_ ≈ 4π*M*_s_ − 2*K*_s_/*M*_s_ *t* ^[^ ^43^ ^]^ and the layer thickness *t* = 4 nm, the interfacial magnetic anisotropy energy density (*K*_s_) is estimated to be giant at the GaAs/CoFe interface (≈ 2.0 erg cm^−2^) but negligibly small at the MgO/CoFe interface (≈0.1 erg cm^−2^). It has been well established that *K*_s_ of magnetic interfaces originates from spin‐orbit coupling (SOC)‐enhanced perpendicular orbital magnetic moments localized at the first ferromagnetic atomic layer adjacent to the interface.^[^ ^44^, ^45^ ^]^ Bruno's tight‐binding model, which has been validated by experiments for a magnetic interface with the SOC strength (*ζ*_SO_) << the bandwidth of the FM,^[^ ^45^, ^46^, ^47^, ^48^ ^]^ predicts *K*_s_ ∝ *ζ*_SO_∆*m*_orb_, where ∆*m*_orb_ is the orbital moment anisotropy of the interface. Given that ∆*m*_orb_ of the GaAs/CoFe interface (∆*m*_orb_≈ 0.085 *µ*_B_) is only slightly greater than that of MgO/CoFe interface (∆*m*_orb_≈ 0.056 *µ*_B_, see Section S2 in the Supporting Information) as suggested by X‐ray magnetic circular dichroism experiments,^[^ ^49^, ^50^, ^51^, ^52^, ^53^ ^]^ the 20 times greater *K*_s_ of the GaAs/CoFe interface than the MgO/CoFe interface reveals considerably stronger interfacial SOC than the MgO/CoFe interface. Therefore, the observation that the sin2*φ* signal is much stronger for the MgO/CoFe than for the GaAs/CoFe strongly disagrees with the previous speculation that the sin2*φ* second HHV signal arose from an interfacial SOC effect.^[^ ^30^ ^]^

**Table 1:** Summary of sample parameters including the resistivity *ρ_xx_*, the effective magnetic anisotropy field *H*_k_, the interfacial perpendicular magnetic anisotropy energy density *K*_s_, the anomalous Nernst voltage *V*_ANE_, the planar Nernst voltage *V*_PNE_, and the planar Hall voltage *V*_PHE_ for the MgO/CoFe[110], GaAs/CoFe[110], Si/SiO_2_/MgO 2 nm/Py 9.4 nm, Si/SiO_2_/MgO 2 nm/FeCoB 9.6 nm, Si/SiO_2_/MgO 2 nm/Co 9.3 nm. The sinusoidal electric field *E* = 6.67 × 10^4^ V cm^−1^

	MgO/CoFe [110]	GaAs/CoFe [110]	Py	Co	FeCoB
*ρ_xx_* [µΩ cm]	46	63	33	36	130
*H*_k_ [kOe]	16.6	6.8	7.3	14.8	11.2
*M*_s_ [emu cm^−3^]	1380	1220	–	–	–
*K*_s_ [erg cm^−2^]	0.1	2.0	–	–	–
*V*_ANE_ [µV]	−0.3	−0.1	−1.9	−2.8	−7.7
*V*_PNE_ [µV]	5.6	0.4	1.0	0.6	0.1
*V*_PHE_ [mV]	3.2	1.7	2.5	1.7	0.4

**Figure 5:**
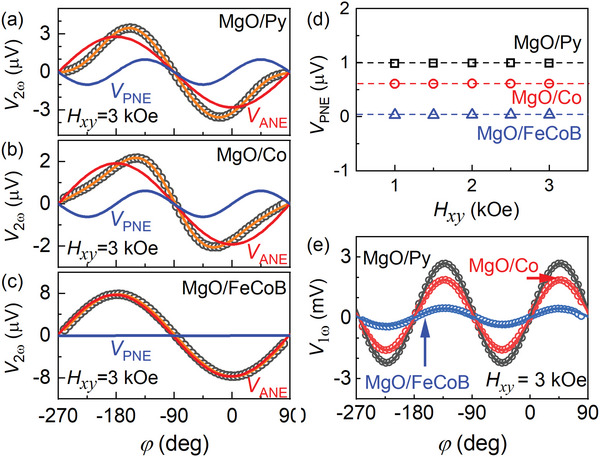
In‐plane angle dependence of the second harmonic Hall voltage for the left crosses of two‐cross Hall bar devices of the sputter‐deposited polycrystalline samples of a) MgO 2 nm/Py 9.4 nm, b) MgO 2 nm/Co 9.3 nm, and c) MgO 2 nm/CoFeB 9.6 nm under an electric field of 66.7 kV m^−1^ and an applied in‐plane magnetic field (*H_xy_*) of 3 kOe. d) Independence of the planar Nernst voltage (V_PNE_) on *H_xy_*. e) In‐plane angle dependence of the first harmonic Hall voltage. Solid curves represent fits of the data to Equation (2) in a–c) and to Equation (1) in (e). The dashed lines in (d) are to guide the eyes. In (c), the curves in orange (the sum signal) and in red (the anomalous Nernst signal) nearly overlap due to the small *V*_PNE_ of the FeCoB (in blue).

Finally, we show in **Figure** **5**a,d that similar sin2*φ* second HHV signals are also observed in control polycrystalline ferromagnetic layers of Py, Co, and FeCoB sputter‐deposited on MgO‐buffered Si/SiO_2_ substrates, which is in contrary to an origin of a low‐symmetry interface of the CoFe single crystal.^[^ ^30^ ^]^ Importantly, for all the samples studied in this work, the magnitude of *V*_PNE_ scales closely with the planar Hall voltage (see Figure 5d,e, and Table 1). For example, *V*_PNE_ (Py) > *V*_PNE_ (Co) > *V*_PNE_ (FeCoB) and *V*_PHE_(Py) > *V*_PHE_(Co) > *V*_PHE_(FeCoB). This is in excellent agreement with the fact that the PNE arises from the same spin‐dependent scattering that generates the AMR and the planar Hall effect.^[^ ^54^, ^55^ ^]^

These observations provide unambiguous evidence that the sin2*φ* second HHV signal arises from the PNE induced by the longitudinal thermal gradient naturally generated due to non‐uniform thermal dissipation across Hall bars and that it is irrelevant to any spin currents.

### 2.3 Anisotropy of the Planar Hall Effect

The PNE is usually known as an isotropic effect in magnetic materials.^[^ ^54^, ^55^, ^56^ ^]^ So far, there has been no report of an anisotropic PNE in any materials. Here, we demonstrate that the PNE in the single crystalline CoFe(001) films has a strong fourfold anisotropy and varies dramatically with crystalline orientation.

**Figure 6:**
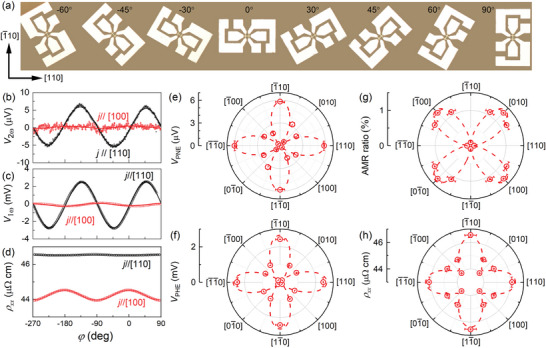
Anisotropic planar Nernst effect in the MgO/CoFe sample. a) Optical microscopy image of the Hall bar devices with different orientations relative to the CoFe [110]. In‐plane angle dependence of b) second harmonic Hall voltage, c) first harmonic Hall voltage, and d) resistivity for current flow along CoFe [110] and [100] orientations. Polar plot of the dependence on the crystal orientation of e) the planar Nernst voltage, f) the planar Hall voltage, g) the AMR ratio, and h) resistivity for the MgO/CoFe single‐crystal sample.

We use Hall bars with the current channel along different orientations of the CoFe crystal (**Figure** **6**a) to measure the anisotropy of *V*_PNE_. In Figure 6b,d, *V*_PNE_, the magnitude of the sin2*φ* second HHV signal is strongly dependent on the crystalline orientation and maximizes when the electric field *E*, thus the current, is applied along the [110], [$\bar{1}$10], [$\bar{1}\bar{1}$0], and [1$\bar{1}$0] orientations of the CoFe but almost vanishes when the current is along the [100], [$\bar{1}$00], [010], and [0$\bar{1}$0] orientations. The strong anisotropy of the PNE is a striking observation because the PNE is for the first time identified to be anisotropic.

To understand the microscopic mechanism of the strong anisotropy of the PNE in CoFe, we measure the resistivity, the AMR ratio, and the planar Hall voltage of the same devices, with the results summarized in Figure 6c,d,f–h. The planar Hall voltage (Figure 6f) has the same dependence on the crystalline orientation as the planar Nernst voltage (Figure 6e), which is consistent with the planar Hall effect and the PNE sharing the same microscopic mechanism.^[^ ^54^, ^55^ ^]^ The planar Hall voltage is reciprocal to the AMR ratio (Figure 6g). As indicated by the first‐principles calculation,^[^ ^31^, ^32^ ^]^ the intrinsic mechanism of the crystal orientation dependence of the AMR in the bcc Co_50_Fe_50_ is the variation of the band crossing. Therefore, the strong anisotropy of the PNE in CoFe single crystal should be attributed to the tuning of the intrinsic band crossing by the crystal orientation. As shown in Figure 6h, there is also a fourfold anisotropy in the resistivity, suggesting a lowered symmetry in the electron scattering in the bulk of the CoFe.

### 2.4 Impacts of the Planar Nernst Effect

**Figure 7:**
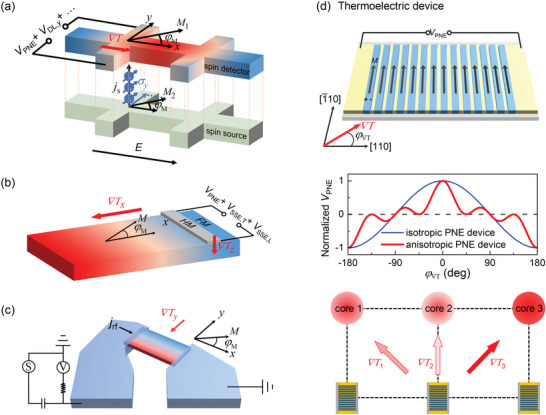
Impacts of the anisotropic planar Nernst effect. a) Entanglement of the planar Nernst voltage with dampinglike torque signals of transverse spins generated by the anomalous Hall effect of an adjacent magnetic layer in a harmonic Hall voltage experiment. b) Entanglement of the planar Nernst voltage with the inverse spin Hall voltages in an experiment of transverse and longitudinal spin Seebeck effects. c) Presence of the planar Nernst voltage in a spin‐torque ferromagnetic resonance experiment. d) Device configuration, characteristics, and power harvesting application of the proposed thermoelectric devices based on the anisotropic PNE (see the main text for more discussions). The magnetic nanowires have a magnetically easy axis along the long axis of the nanowires due to shape anisotropy, allowing external magnetic field‐free operation of the device. The isotropic and anisotropic PNE devices have distinct dependences on the thermal gradient orientation ($\varphi_{\nabla T}$). Consequently, the thermal gradients at $\varphi_{\nabla T}$ = ±45^°^, that is, ∇*T*_1_ and ∇*T*_3_ in the bottom panel of (d), contribute no signal to the anisotropic PNE device, leaving the device sensing only the thermal gradient ∇*T*_2_ at $\varphi_{\nabla T}$ = 0^°^. However, the isotropic PNE device is affected by thermal gradients from all orientations.

Our discovery of the strong, anisotropic PNE can have widespread scientific and technological impacts. First, our results suggest that the planar Nernst voltage can be significant within Hall bars and magnetic strips and thus should be carefully separated, for example, via dependences on the magnetization angle (*φ_M_*), thermal gradient angle ($\varphi_{\nabla T}$), magnetic field strength, and/or temperature gradient, for accurate analyses of a variety of key spintronic experiments. In HHV measurements on Hall bar devices, the PNE, with the electric field ***E***_PNE_ ∝ ***M*** × (***M*** × ∇ ***T***), manifests as a sin2*φ_M_* dependent second harmonic Hall voltage (Figure 2d and Figure 4a) that will entangle with that signal of the dampinglike torque of the transverse spins (*V*_DL,y_) generated by the anomalous Hall effect in another magnetic layer (**Figure** **7**a). In spin Seebeck experiments, *V*_PHE_ will also add to the inverse spin Hall voltages from the longitudinal and transverse spin Seebeck effects (*V*_SEE,L_ and *V*_SEE,T_, see Figure 7b). In spin‐torque ferromagnetic resonance experiments on magnetic strips with asymmetric current spreading^[^ ^35^ ^]^ (e.g., the two‐terminal devices in Figure 7c), the PNE contributes additional symmetric intermixing voltage signals through the association with the dampinglike torques of longitudinal spins ($S_{\mathrm{DL},x}^{\mathrm{PNE}}$sin^2^ *φ_M_* sin2*φ_M_*), transverse spins ($S_{\mathrm{DL},y}^{\mathrm{PNE}}$cos^2^ *φ_M_* sin2*φ_M_*), and perpendicular spins ($S_{\mathrm{DL},z}^{\mathrm{PNE}}$ sin2*φ_M_*). Importantly, these PNE‐associated signals are expected to have potential crystalline anisotropy, which may simply arise from the anisotropy of the PNE, without any tie to anisotropic generation of spin polarizations due to low crystal symmetry.

From the point of view of technological applications, the discovery of the strong, anisotropy PNE opens a promising avenue for the development of PNE‐based thermoelectric technologies. As an example, we propose in Figure 7d a thermoelectric device structure comprising many pairs of in‐plane magnetized nanowires that are electrically connected in series, for example, along the CoFe [110] that has strong PNE. In such a structure, the sensitivity is enhanced by a factor of the pair number because this structure increases the effective length of the magnetic material and thus the *V*_PNE_ output. The large in‐plane magnetization of CoFe can also stabilize an in‐plane easy axis along the long axis of the CoFe nanowires via shape anisotropy, enabling magnetic‐field‐free operation of the thermoelectric devices. Such PNE devices can function as thermoelectric batteries for energy harvesting and thermal‐gradient sensors. For example, it can sensitively locate the thermal nonuniformity in a material, device, or circuit by sensing the thermal gradient. It is straightforward to derive that the *V*_PNE_ output of the proposed thermoelectric device in Figure 7d has a cos^2^2$\varphi_{\nabla T}$cos$\varphi_{\nabla T}$ dependence for the CoFe device with anisotropic PNE, which is in sharp contrast to the cos$\varphi_{\nabla T}$ dependence of a similar device made of isotropic PNE material. When such thermoelectric PNE devices are used to monitor arrays of working units such as computing cores in the bottom panel of Figure 7d, the anisotropic PNE device would be sensitive only to the nearest computing core at $\varphi_{\nabla T}$ = 0^°^, but unaffected by the second neighboring cores that are at $\varphi_{\nabla T}$ = ±45^°^. Consequently, the anisotropic PNE device can accurately locate the heavily and lightly working cores for task distribution analysis, which is advantageous over the conventional isotropic PNE device.

## 3 Conclusion

We have presented unambiguous evidence that there is no sizable spin current generation from the bulk or interfaces of CoFe single crystal, even when the interface has very strong spin‐orbit coupling (as in the case of the GaAs/CoFe sample). We verify that the sin2*φ* second harmonic Hall voltage, which was previously assumed to signify the Dresselhaus‐like spin current in the same materials,^[^ ^30^ ^]^ is *unrelated* to any spin currents. Instead, it is the PNE voltage induced by the temperature gradient within the sample. We have shown that such sin2*φ* second harmonic Hall voltage is independent of the applied magnetic field, inversely correlated to the heat capacity of the substrates and overlayers, quadratic in the electric field, thus current, and also appears in polycrystalline ferromagnets. Strikingly, we have for the first time observed that the PNE in a ferromagnet is strongly anisotropic and varies in strength with the crystalline orientation of single crystals in analogue to the planar Nernst effect. The strong, anisotropic PNE not only points to a critical need to carefully separate the planar Nernst voltage in a variety of spintronic experiments (e.g., harmonic Hall voltage, spin Seebeck effect, and spin‐orbit torque ferromagnets) but also opens a new avenue for the development of thermoelectric battery and sensor technologies based on anisotropic PNE. Our findings will stimulate the exploration of anisotropic PNE and its impacts in various magnetic systems.

## Conflict of Interest

The authors declare no conflict of interest.

## Supporting information

Supporting Information

## Data Availability

The data that support the findings of this study are available from the corresponding author upon reasonable request.
