# Supplementary material for: Absence of Spin‐Orbit Torque and Discovery of Anisotropic Planar Nernst Effect in CoFe Single Crystal
Source: Adv Sci (Weinh). 2023 Jul 23;10(27):2301409. doi: 10.1002/advs.202301409 (PMC10520638; doi:10.1002/advs.202301409)
Supplement: Supplementary file 1 — Supporting Information [file ADVS-10-2301409-s001.pdf]

ADVANCED SCIENCE
Open Access

Supporting Information

for *Adv. Sci.*, DOI 10.1002/advs.202301409

Absence of Spin-Orbit Torque and Discovery of Anisotropic Planar Nernst Effect in CoFe Single Crystal

*Qianbiao Liu, Xin Lin and Lijun Zhu**

Supporting Information for

Absence of Spin-Orbit Torque and Discovery of Anisotropic Planar Nernst Effect in CoFe Single Crystal

Qianbiao Liu,[1] Xin Lin,[1,2] Lijun Zhu[1,2]*

1. State Key Laboratory for Superlattices and Microstructures, Institute of Semiconductors, Chinese Academy of Sciences, Beijing 100083, China

2. College of Materials Science and Opto-Electronic Technology, University of Chinese Academy of Sciences, Beijing 100049, China

**ljzhu@semi.ac.cn*

1. Comment on the control experiment of Adv. Funct. Mater. 32, 2204212 (2022)

In the main text, we have unambiguously established that the sin2φ second harmonic Hall signals of the CoFe and other ferromagnets are the planar Nernst voltage (V_{ANE}) associated with the in-plane temperature gradient. Here, we further clarify that there is NOT any indication against the planar-Nernst-voltage interpretation of the sin2φ signal in the previous paper (Wang *et al*., Adv. Funct. Mater. 32, 2204212 (2022)[1]). Reference [1] asserted that the thermal interpretation was not the mechanism because the entire signal of a three-cross Hall bar device of a MgO/CoFe 2 nm sample was the "same" for different locations of the Hall cross on the Hall bar device, without any quantitative specification of the relative strength of the sin2φ signals.

To be objective, the control experiment of ref. [1] deliberately chose the "worst" 2 nm control sample which has the lowest sin2φ signal relative to the anomalous Nernst signals (*i.e*., the cosφ signals, see Fig. S1a-1f). At the same, Ref. [1] considerably lowered the current flow within the device in the control measurement, in the attempt to disprove the presence of a thermal effect. As can be seen from Fig. S1a and Fig. S2b, the total second harmonic Hall signal is 12.9 μV during the torque measurement but lowered by a factor of 6.5 to 2.0 μV in the control experiment. All these selections were made such that the sin2φ signal and its thermoelectric origin were as hidden as possible in Ref. [1].

Even so, the sin2φ signal in Ref. [1] varies significantly with the location of the cross on the three-cross Hall bar device, in good agreement with the planar Nernst effect. As we have separated in Fig. S2b and 2c, the sin2φ signal for the Hall cross 2 (in the left part of the Hall bar) of the Ref. [1] is 40% smaller than the Hall cross 1 (in the center of the Hall bar) because of the higher temperature at the center Hall cross than at the left Hall cross. According to the Mott relation [2], the Nernst conductivity (α_{xy}) increases significantly with temperature (T) below the Curie temperature, $\alpha_{xy} \approx \pi^2 k_B^2 T/3e\frac{d\sigma_{xy}}{dE}$, where k_B is the Boltzmann constant, e the elementary charge, σ_{xy} the anomalous Hall conductivity, and E the energy.

Therefore, the sin2φ signal of the Hall crosses in Ref. [1] is also due to the anomalous Nernst effect. While it is not possible to tell any pattern/contact alignment asymmetry from the optical microscopy image of the device in Fig. S2a due to its low resolution, it is not surprising for microstructures to have unintentional symmetry imperfection [3,4]. For example, Ref. [3] has reported non-uniform current distribution in nominally

symmetric Hall bars and spin-torque ferromagnetic resonance microstrips; ref. [4] has reported longitudinal temperature gradient-induced planar Nernst signal in their nominally symmetric NiFe Hall crosses.

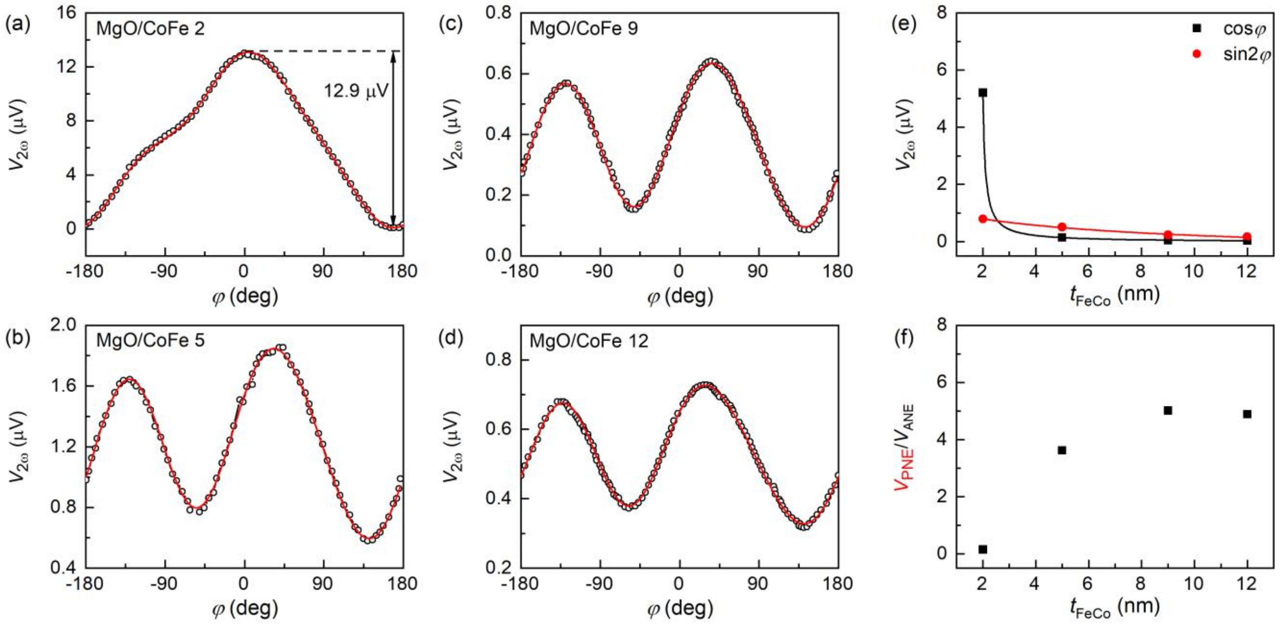

Figure S1. Dependence on the in-plane field angle of the second harmonic Hall voltage for MgO/CoFe samples with CoFe thickness of (a) 2 nm, (b) 5 nm, (c) 9 nm, and (d) 12 nm. (e) The magnitudes of the cosφ and the sin2φ signals (i.e., the anomalous Nernst voltage V_{ANE} and the planar Nernst voltage V_{PNE}) and (f) their relative strength for the MgO/CoFe samples. The data in (a)-(d) are adapted directly from Ref. [1], and the data in (e) and (f) are from fits of the data in (a)-(d). As can be seen in (e) and (f), the 2 nm sample has the smallest relative strength of the sin2φ signals.

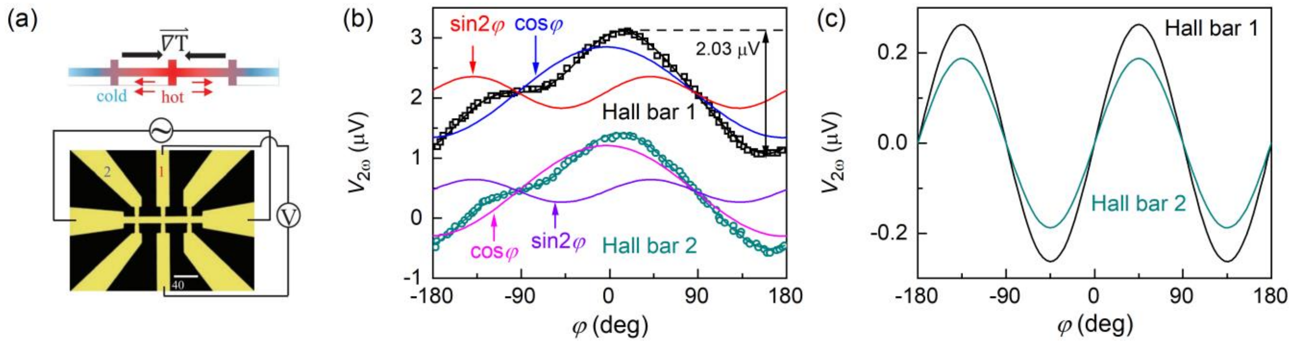

Figure S2. (a) Schematic of the in-plane temperature gradient for a perfectly symmetric three-cross Hall bar device and the optical microscopy image of a Hall bar device for the control experiment in Ref. [1], with the number 1 and 2 marking two Hall crosses. (b) In-plane field angle dependence of the second harmonic Hall voltage $V_{2\omega}$ for the Hall cross 1 (black data) and Hall cross 2 (cyan data) of a MgO/CoFe 2 nm/AlO_x control sample in Ref. [1]. (c) Comparison of the sin2φ signals of Hall cross 1 (black data) and Hall cross 2 (cyan data). In (b) the solid black and cyan curves represent fits of the data to Eq. (2) in the main text. The data in (a) and (b) are adapted directly from Ref. [1].

2. Average orbital magnetic moment anisotropy of GaAs/CoFe and MgO/CoFe interfaces

According to Bruno's tight-binding model, the interfacial perpendicular magnetic anisotropy energy density (K_s) for a certain FM interface is proportional to the product of the SOC (ζ) and the orbital moment anisotropy (Δm_{orb}) of the interface, i.e., $K_s \propto \zeta\Delta m_{orb}$. The value of Δm_{orb} is given by the difference between the perpendicular and the in-plane orbital magnetic moments, $m_{orb,\perp}$ and $m_{orb,||}$. The values of $m_{orb,\perp}$ and $m_{orb,||}$ of an interface can be determined by XMCD measurements at normal and grazing incident configurations, respectively. In general, $m_{orb,\perp}$ of the ferromagnetic interface is greater than $m_{orb,||}$ in the thin limit and reduces inversely with the layer thickness towards the magnitude of $m_{orb,||}$ given that the crystal structure does not change significantly with the layer thickness.

As indicated by previous XMCD studies (see a summary in Table S1), $\Delta m_{orb} \approx 0.022\ \mu_B$ for the MgO/Fe (1 ML) interface [5,6] and 0.09 μ_B for the MgO/Co (1ML) interface [7] grown at room temperature, yielding an average value of $\Delta m_{orb} \approx 0.056\ \mu_B$ for the MgO/$Co_{0.5}Fe_{0.5}$ interface; $\Delta m_{orb} \approx 0.08\ \mu_B$ for the GaAs/Fe (1 ML) interface [8] and 0.09 μ_B for the GaAs/Co (1ML) interface [9] grown at room temperature, yielding an average value of $\Delta m_{orb} \approx 0.085$ for the GaAs/$Co_{0.5}Fe_{0.5}$ interface. Therefore, the average orbital magnetic moment anisotropy is only slightly stronger for the GaAs/$Co_{0.5}Fe_{0.5}$ interface than for the MgO/$Co_{0.5}Fe_{0.5}$ interface.

Table S1. Orbital magnetic moments of Co and Fe at the MgO and GaAs interfaces from XMCD measurements.

	$m_{orb,\perp}$	$m_{orb,\|\|}$	Δm_{orb}	Note
MgO/Fe (1 ML)	0.207 [5]	0.185 [5]	0.022	Consistent with Ref. [6]
MgO/Co (1ML)	>0.16 [8]	0.07 [8]	0.09	We do not use the $m_{orb,\perp}$ data of Fe in [8] because that for the Ta/Fe/MgO includes contributions from both Ta/Fe and Fe/MgO interfaces and is thus larger than reported for Fe/MgO [5,6]. As indicated by the two-magnon scattering study [10], the interfacial SOC effect is negligible at the Ta/Co interface but significant at the Ta/Fe interface.
GaAs/Fe (1ML)	0.23 [7]	0.15 [7]	0.08	
GaAs/Co (1ML)	0.25 [9]	0.16 [9]	0.09	

Reference

[1] W. Wang, Z. Yan, Y. Cao, C. Gao, Z. Shi, M. Si, J. Cao, L. Xi, D. Yang, and D. Xue, Generation and Detection of Dresselhaus-Like Spin Current in a Single-Crystal Ferromagnetic Metal, Adv. Funct. Mater. 32, 2204212 (2022).

[2] Y. Pu, D. Chiba, F. Matsukura, H. Ohno, and J. Shi, Mott Relation for Anomalous Hall and Nernst Effects in $Ga_{1-x}Mn_xAs$ Ferromagnetic Semiconductors, Phys. Rev. Lett. 101, 117208 (2008).

[3] J. Zhou, X. Shu, W. Lin, D. F. Shao, S. Chen, L. Liu, P. Yang, E. Y. Tsymbal, J. Chen, Modulation of Spin–Orbit Torque from $SrRuO_3$ by Epitaxial-Strain-Induced Octahedral Rotation, Adv. Mater. 33, 2007114 (2021).

[4] Q. Liu, and L. Zhu, Current-induced perpendicular effective magnetic field in magnetic heterostructures, Appl. Phys. Rev. 9, 041401 (2022).

[5] K. Mamiya, T. Koide, Y. Ishida, Y. Osafune, A. Fujimori, Y. Suzuki, T. Katayama, S. Yuasa, Angle-resolved soft X-ray magnetic circular dichroism in a monatomic Fe layer facing an MgO(001) tunnel barrier, Radiation Physics & Chemistry, 75, 1872 (2006).

[6] S. Sakamoto, E. Jackson, T. Kawabe, T. Tsukahara, Y. Kotani, K. Toyoki, E. Minamitani, Y. Miura, T. Nakamura, A. Hirohata, and S. Miw, Control of perpendicular magnetic anisotropy at the Fe/MgO interface by phthalocyanine insertion, Phys. Rev. B 105, 184414 (2022).

[7] J. S. Claydon, Y. B. Xu, M. Tselepi, J. A. C. Bland, and G. van der Laan, Direct Observation of a Bulklike Spin Moment at the Fe/GaAs (100)−4×6 Interface, Phys. Rev. Lett. 93, 037206 (2004).

[8] S. Kanai, M. Tsujikawa, Y. Miura, M. Shirai, F. Matsukura, and H. Ohno, Magnetic anisotropy in Ta/CoFeB/MgO investigated by x-ray magnetic circular dichroism and first-principles calculation, Appl. Phys. Lett. 105, 222409 (2014).
[9] L. Giovanelli, G. Panaccione, G. Rossi, M. Fabrizioli, C.-S. Tian, P. L. Gastelois, J. Fujii, and C. H. Back, Layer-selective spectroscopy of Fe⁄GaAs(001): Influence of the interface on the magnetic properties, Phys. Rev. B 72, 045221 (2005).
[10] L. Zhu, L. Zhu, D.C. Ralph, and R.A. Buhrman, Origin of Strong Two-Magnon Scattering in Heavy-Metal/Ferromagnet/Oxide Heterostructures, Phys. Rev. Applied 13, 034038 (2020).
